# Sacroiliac and spine imaging in spondyloarthritis: Does phenotype or sex matter?

**DOI:** 10.1186/s42358-024-00411-w

**Published:** 2024-09-10

**Authors:** Gabriel Caetano Pereira, Natalia Pereira Machado, André Francisco Gomes, Rodrigo Luppino Assad, Fabio Henrique Carneiro, Valderílio Feijó Azevedo

**Affiliations:** 1 UFPR, Curitiba, Brazil; 2 CHC-UFPR/EBSERH, Curitiba, Brazil; 3 UFPR, Curitiba, Brazil; 4 HC-FMUSP-RP, Ribeirão Preto, Brazil

**Keywords:** Axial spondyloarthritis, Axial psoriatic arthritis, Sacroiliac magnetic resonance imaging, Sacroiliitis

## Abstract

**Background:**

Spondyloarthritis (SpA) encompasses a spectrum of immune-mediated inflammatory conditions primarily affecting the axial skeleton, including sacroiliitis and spondylitis, each with distinct features. This study aimed to investigate imaging disparities, focusing on sacroiliac magnetic resonance and spine radiography, across phenotypes and between males and females in axial SpA.

**Method:**

A cross-sectional study was conducted to assess clinical data, laboratory findings, magnetic resonance imaging (MRI) scores of sacroiliac joints using the Spondyloarthritis Research Consortium of Canada (SPARCC) and Sacroiliac Joint Structural Score (SSS), and cervical and lumbar spine radiographs utilizing the Modified Stoke Ankylosing Spondylitis Spine Score (mSASSS). The study aimed to compare these parameters between two groups: axial spondyloarthritis (axSpA, radiographic and non-radiographic) and axial psoriatic arthritis (axPsA), as well as between males and females.

**Results:**

Ninety-four patients were included, with 62 patients in the axSpA group and 32 patients in the axPsA group. There were no differences in disease activity, mobility, radiographic damage in the spine (Modified Stoke Ankylosing Spondylitis Spine Score– mSASSS), or sacroiliac magnetic resonance imaging (MRI) scores (Spondyloarthritis Research Consortium of Canada Magnetic Resonance Imaging Index - SPARCC and Sacroiliac Joint Structural Score - SSS) between the two phenotypes. Regarding sex, in imaging exams, men had higher mSASSS (*p* = 0.008), SSS (*p* = 0.001), and fat metaplasia (MG) score based on SSS (*p* = 0.001), while women had significantly higher SPARCC scores (*p* = 0.039). In the male group, the presence of HLA-B27 allele had an impact on more structural lesions on MRI (SSS), *p* = 0.013.

**Conclusion:**

In this study, imaging of sacroiliac joints and spine in patients with axial SpA did not show differences in phenotypes but did reveal differences based on sex, which may have an impact on future diagnostic recommendations. Further studies are needed to confirm these findings.

## Introduction

The term spondyloarthritis (SpA) encompasses a group of chronic inflammatory arthropathies that share genetic and clinical aspects, including ankylosing spondylitis (AS), non-radiographic axial spondyloarthritis (nr-axSpA), psoriatic arthritis (PsA), reactive arthritis, enteropathic spondyloarthritis, and undifferentiated spondyloarthritis [1, 2]. Unlike AS, axial involvement in PsA has a heterogeneous presentation, with a group of patients being completely asymptomatic and others experiencing axial pain, either mechanical or inflammatory in nature. In addition to the differences in clinical presentation between AS and axial PsA, radiographic changes also differ; in PsA, asymmetry in sacroiliac involvement, coarser syndesmophyte formation with an angulation > 45° (referred to as para-syndesmophytes), isolated involvement of the lumbar spine without sacroiliitis, and frequently prevalent and often isolated involvement of the cervical spine have been observed [3–5].

A Brazilian study evaluated magnetic resonance imaging (MRI) of the sacroiliac joints in PsA patients, regardless of symptoms, and found a prevalence of sacroiliitis of 37.8%, with 47% being unilateral. The most prevalent MRI findings were bone marrow edema (BME), enthesitis, erosion (ER), and fat metaplasia (FAT). Additionally, longer psoriasis duration was predictive of structural lesions [6]. On the other hand, there are few studies comparing MRI findings of sacroiliac joints between axial SpA and axial PsA.

In addition to the differences between phenotypes, sex also appears to influence the clinical manifestations and imaging findings of spondyloarthritis. Women present more peripheral joint involvement, pain, disease activity, and diagnostic delay, while men exhibit greater radiographic damage and higher levels of inflammatory markers [7, 8]. Some of these differences may be related to genetic factors and sex hormones influencing the immune response. These biological mechanisms may contribute to different disease manifestations, perceptions, and treatment responses in men and women with SpA [9].

The aim of this study was to compare the scores obtained from magnetic resonance imaging (MRI) of the sacroiliac joint, specifically using the Spondyloarthritis Research Consortium of Canada (SPARCC) and the Sacroiliac Joint Structural Score (SSS), alongside radiographs of the cervical and lumbar spine, assessed using the Modified Stoke Ankylosing Spondylitis Spine Score (mSASSS). We sought to examine any variations in these scores among patients with axial spondyloarthritis (both radiographic and non-radiographic subtypes) and axial psoriatic arthritis, with an additional focus on assessing potential differences based on sex. Furthermore, our objective included correlating these imaging findings with demographic and clinical characteristics.

## Methods

### Clinical assessment

This was a cross-sectional study in which two groups of patients were selected, with all of them required to meet the criteria for axial spondyloarthritis of the Assessment of Spondyloarthritis International Society criteria (ASAS) [2]. Those patients who also met the Classification Criteria for Psoriatic Arthritis (CASPAR) [10] with axial involvement were allocated to the axPsA group. Axial involvement was defined as the presence of low back pain for more than 3 months and one of the following findings recorded in the medical records: radiographic sacroiliitis according to the New York classification [11] or positive sacroiliac magnetic resonance imaging for axial spondyloarthritis [12].

Exclusion criteria included age under 18 years or concurrent inflammatory rheumatic diseases. Demographic measures (sex, self-reported skin color, age, body mass index - BMI, occupation, smoking information, and physical activity at the time of data collection) and clinical data (disease duration, presence of peripheral arthritis, enthesitis, dactylitis, or extra-musculoskeletal manifestations at any time during the disease) were evaluated. Manual labor was defined predominantly hand-driven activities, often involving the direct manipulation of tools and materials without significant automation. Information was collected on the following validated assessment instruments of disease activity: Bath Ankylosing Spondylitis Disease Activity Index (BASDAI) [13], Axial Spondyloarthritis Disease Activity Score - C- Reactive Protein (ASDAS-CRP) [14]. Function and mobility were analyzed respectively by Bath Ankylosing Spondylitis Functional Index (BASFI) [15] and Bath Ankylosing Spondylitis Metrology Index (BASMI) [16]. The psoriatic arthritis activity indices used were: Psoriasis Area Severity Index (PASI) [17] and Body Surface Area (BSA) [18], for psoriasis and Disease Activity Index for Psoriatic Arthritis (DAPSA) [19] for articular involvement. Additional data were collected on complementary tests, such as the presence of HLA-B27 allele and imaging examination. The patients registered as having fibromyalgia were identified through medical records, and no classification criteria were applied for this condition.

### Radiography imaging

For radiographic imaging, the Modified Stoke Ankylosing Spondylitis Spine Score (mSASSS) [20] was employed, calculated from lateral cervical and lumbar spine radiographs. The mSASSS was performed on patients with the radiographic form of axial spondyloarthritis and on patients with axial psoriatic arthritis. This method is the preferred choice endorsed by Outcome Measures for Arthritis in Clinical Trials (OMERACT) [21]. This involved measuring anterior angles from the inferior endplate of C2 to the superior endplate of T1 and from the inferior endplate of T12 to the superior endplate of S1. Erosion, squaring, shiny corner, syndesmophyte, or bridging were considered, and each was scored from 0 to 3. In cases where certain vertebrae were not visible due to artifacts or imaging technique limitations, an average score was calculated for the missing vertebrae. Samples with three or more vertebrae not visualized were excluded. Two rheumatologists, blinded to clinical information (NPM and RLA), performed this assessment.

### Magnetic resonance imaging

Regarding magnetic resonance imaging (MRI), the SPARCC score [22] and the Sacroiliac Joint Structural Score (SSS) [23] were calculated. The SPARCC score assessed active inflammatory lesions in the sacroiliac joints using T2-weighted sequences with fat suppression or STIR. Scoring was conducted on 6 consecutive coronal slices per joint, with each joint divided into 4 quadrants. Increased signal presence in these quadrants was scored dichotomously, (1, increased signal and 0, normal signal). The maximum score for abnormal signal in the 2 joints of 1 coronal slice is 8. Quadrants that include a lesion exhibiting intense signal and/or depth > 1 cm from the articular surface receive an additional 1 point for each of these findings. Therefore, each slice has a maximum score of 12, with the total maximum (6 slices) being 72. The SSS evaluated structural lesions in the sacroiliac joints based on T1-weighted sequences, scoring fat metaplasia, erosion, backfill, and ankylosis. Scoring was performed on 5 coronal slices anterior to the transitional slice, identified by the appearance of the ligamentous portion (the most posterior part of the joint). The scoring ranges are: for fat metaplasia (FM) (0–40), for erosion (0–40), for backfill (BF) (0–20), and for ankylosis (0–20), thus, the maximum score is 120. To score erosion and fat metaplasia, each joint is divided into 4 quadrants, so each slice has 8 quadrants. To score ankylosis and backfill, each joint is divided into 2 halves (upper and lower). Assessments were carried out by a rheumatologist (NPM) and a radiologist (AFG), both trained in the method and blinded to clinical information. Clinical and laboratory information collected were recorded within a range of up to 6 months before or after the MRI scan and up to 1 year before or after the radiographic examinations.

### Statistical analysis

The data were analyzed descriptively, with categorical variables presented as absolute and relative frequencies, and numerical variables as mean and standard deviation. The normality of variables was assessed using the Kolmogorov-Smirnov test. The Fisher’s exact test was used to test the association between categorical variables, and the Student’s t-test and Mann-Whitney U test were used for normally and non-normally distributed numerical variables, respectively. Spearman correlations were conducted between numerical variables. Imaging scores mSASSS, SSS, and SPARCC were defined as dependent variables. To investigate the association between the independent variables age, sex, BMI, disease duration, HLAB27 positivity, manual labor, smoking, BASDAI, ASDAS-CRP and dependent variables, linear regression models were fitted. Subsequently, multivariate models were adjusted including independent variables that showed statistical significance and tendency to significance in the univariate models. In all models, the Student’s t-test was used to evaluate the significance of the independent variables. Intraclass correlation (ICC) analyses were performed for the readings of mSASSS, SPARCC, and SSS. The IBM SPSS Statistics version 23 software was employed for the analysis, and p-values < 0.05 were deemed significant.

## Results

A total of 94 patients were included in the study, evaluated between 2015 and 2022, with 62 patients in the axial spondyloarthritis (axSpA) group and 32 patients in the axial psoriatic arthritis (axPsA) group, who signed the informed consent form. Among the axSpA patients, 24 presented with the non-radiographic form, while 38 exhibited the radiographic form. Of these 94 patients, 56 had magnetic resonance imaging (MRI) of the sacroiliac joints (35 men and 21 women), and 58 had cervical and lumbar spine radiographs (38 men and 20 women). The intraclass correlation coefficient (ICC) for mSASSS was 0.763 (95% CI: 0.607–0.862), for the SPARCC and SSS scores were 0.879 (95% CI: 0.790–0.932) and 0.905 (95% CI: 0.715–0.959), respectively. The majority of the total group were men (62.8%), white (82.8%), and overweight (mean BMI of 28.6 kg/m²). Other demographic characteristics of the sample are shown in Table 1.

**Table 1:** Clinical and demographic characteristics of the total patient sample, according to phenotype

Variables	axSpA (*N* = 62)	axPsA (*N* = 32)	Total (*N* = 94)	*p*
Age, years	44,12 (± 8,3)	51,10 (± 12,7)	48,40 (± 10,6)	**0**,**010**
Male sex, n %	37 (59,7%)	22 (68,8%)	59 (62,8%)	0,389
Whites, n %	52 (83,9%)	25 (80,6%)	77 (82,8%)	0,698
BMI, kg/m²	28,1 (± 5,1)	27,9 (± 4,8)	28,6 (± 5,1)	0,492
Disease duration, years	15,9 (± 10,7)	15,7 (± 8,3)	15,8 (± 9,8)	0,816
ASDAS-CRP	2,7 (± 1,1)	3,2 (± 1,0)	2,9 (± 1,1)	0,097
BASDAI	4,4 (± 2,0)	4,8 (± 2,1)	4,5 (± 2,0)	0,275
BASFI	4,3 (± 2,7)	8,1 (± 14,4)	5,8 (± 8,9)	0,257
BASMI	3,8 (± 1,5)	5,9 (± 9,2)	5,5 (± 8,8)	0,605
Regular physical activity, n%	12 (19,7%)	7 (21,5%)	19 (20,4%)	0,802
HLAB27 positive, n %	43 (71,7%)	8 (30,8%)	51 (59,3%)	**< 0**,**001**
Manual labor, n %	18 (33,3%)	5 (16,7%)	23 (27,4%)	0,101
Smoking, n%	8 (14,3%)	3 (10,3%)	11 (12,9%)	0,742
Psoriasis, n%	3 (4,8%)	30 (93,8%)	33 (35,1%)	**< 0**,**001**
Nail psoriasis, n%	1 (1,6%)	9 (28,1%)	10 (10,6%)	**< 0**,**001**
DAPSA	-	15,6 (± 15,3)	-	
BSA, %	-	2,9 (± 5,0)	-	
PASI	-	3,4 (± 7,2)	-	
Fibromyalgia, n %	12 (19,4%)	8 (25,0%)	20 (21,3%)	0,526
Uveitis, n %	16 (25,8%)	3 (9,4%)	19 (20,2%)	0,060
Inflammatory bowel disease, n %	4 (6,6%)	0 (0,0%)	4 (4,3%)	0,295
Arthritis, n %	20 (32,3%)	23 (71,9%)	43 (45,7%)	**< 0**,**001**
Enthesitis, n %	32 (53,3%)	12 (37,5%)	44 (47,8%)	0,148
Dactylitis, n %	1 (1,6%)	3 (9,4%)	4 (4,3%)	0,113
Anti-TNF, n %	41 (66,1%)	12 (37,5%)	53 (56,3%)	**0**,**01**
Anti-IL-17, n%	10 (16,1%)	14 (43,7%)	24 (25,5%)	**0**,**002**
Anti-IL12/23, n%	0	1 (3,1%)	1 (1,0%)	0,560
NSAIDs, n %	26 (41,9%)	7 (4,57%)	33 (35,1%)	**0**,**026**

*axSpA* axial spondyloarthritis, *axPsA* axial psoriatic arthritis, *BMI* Body Mass Index, *BASDAI* Bath Ankylosing Spondylitis Disease Activity Index, *ASDAS* Axial Spondyloarthritis Disease Activity Score, *BASFI* Bath Ankylosing Spondylitis Functional Index, *BASMI* Bath Ankylosing Spondylitis Metrology Index, *PASI* Psoriasis Area Severity Index, *BSA* Body Surface Area, *DAPSA* Disease Activity index for Psoriatic Arthritis, *axSpA* axial spondyloarthritis, *axPsA* axial psoriatic arthritis, *anti-TNF* anti-tumor necrosis factor, *anti-IL-17* anti-interleukin 17, *Anti-IL12/23* anti-interleukin 12 and 23, *NSAID* non-steroidal anti-inflammatory drug. Values in bold indicates statistical significance with *p* < 0.05

In the total group, mSASSS was higher in manual labor workers (28.27 ± 18.93; *p* = 0.003) and correlated positively with FM score by the SSS, as well as with age, BMI, and BASMI as described in Table 2. In the multivariate linear regression model with the dependent variable being mSASSS and the independent variables being sex, age, manual labor, only age (*p* = 0,002) and manual labor were associated with mSASSS. The regression results can be better observed in Table 3.

**Table 2:** Correlations between imaging variables in the total group

	mSASSS	SPARCC	SSS	FM
Age	***r*** **= 0,394** ***p*** **= 0,002**	*r*=-0,111 *p* = 0,415	*r*=-0,145 *p* = 0,285	*r* = 0,117 *p* = 0,390
BMI	***r*** **= 0,291** ***p*** **= 0,027**	*r*=-0,213 *p* = 0,118	*r*=-0,089 *p* = 0,519	*r* = 0,080 *p* = 0,562
BASMI	***r*** **= 0,494** ***p*** **= 0,005**	*r* = 0,138 *p* = 0,410	*r* = 0,114 *p* = 0,494	*r* = 0,114 *p* = 0,496
FM	***r*** **= 0,417** ***p*** **= 0,016**	*r*=-0,209 *p* = 0,122	*r* = 0,692* p = < 0,001*	*r* = 1,00
BF	*r*=-0,009 *p* = 0,961	***r*** **= 0,302** ***p*** **= 0,024**	*r* = 0,210 *p* = 0,120	*r* = 0,139 *p* = 0,307
Disease duration	*r* = 0,109 *p* = 0,423	*r*=-0,134 *p* = 0,343	***r*** **= 0,376** ***p = 0***,**006**	***r*** **= 0,302** ***p*** **= 0,029**

*mSASSS* Modified Stoke Ankylosing Spondylitis Spine Score, *SPARCC* Spondyloarthritis Research Consortium of Canada Magnetic Resonance Imaging Index, *SSS* Sacroiliac Joint Structural Score, *FM* Fat Metaplasia, *BMI* Body Mass Index, *BASMI* Bath Ankylosing Spondylitis Metrology Index, *BF* backfill. *FM is part of SSS assessment, so its statistical result was disregarded. Values in bold indicates statistical significance with *p* < 0.05

**Table 3:** Multivariate linear regression models

	**MSASSS**		
Independent variable	*p*	Estimated coefficient (CI 95%)	R^2^ adjusted
Age	**0**,**002**	0,78 (0,31; 1,25)	28,2%
Manual labor	**0**,**016**	11,9 (2,32; 21,6)	
Sex	0,182	-6,32 (-15,7; 3,05)	
	**SSS**		
Sex	**0**,**002**	-10,6 (-19,4; -1,77)	19,3%
HLA-B27 positive	**0**,**014**	9,88 (2,10; 17,7)	
Disease duration	0,559	0,13 (-0,32; 0,58)	
	**SPARCC**		
Sex	**0**,**035**	5,51 (0,41; 10,6)	21,9%
BMI	0,069	-0,48 (-1,00; 0,04)	
Disease duration	0,052	6,94 (-0,05; 13,9)	

*mSASSS* Modified Stoke Ankylosing Spondylitis Spine Score, *SPARCC* Spondyloarthritis Research Consortium of Canada Magnetic Resonance Imaging Index, *SSS* Sacroiliac Joint Structural Score, *BMI* Body Mass Index, *CI* confidence interval. Linear regression model (multivariate), *p* < 0.05 R^2^: coefficient of determination. Values in bold indicates statistical significance with *p* < 0.05

Regarding SSS, a positive correlation with disease duration was observed (Table 2). Additionally, a positive correlation was noted between SPARCC score and BF score by SSS. No correlation was found between clinical findings such as BASDAI, ASDAS-CRP, BASMI or BASFI with sacroiliac MRI findings (Table 2).

### Differences between axSpA and axPsA groups

There was no difference between these two groups concerning radiographic damage in the spine (mSASSS) or the evaluation of sacroiliac MRI using the SSS and SPARCC, but there was a trend towards a higher SSS score in the axSpA group (*p* = 0.068) as shown in Table 4. In the axSpA group, a higher positivity of the HLA-B27 allele and a trend towards more cases of uveitis (*p* = 0.060) were observed. In the axPsA group, there was a higher mean age (*p* = 0.01) and a higher presence of peripheral arthritis (*p* < 0.001). Disease activity, function and mobility were similar in both groups (Table 1). Regarding treatment, there was a higher use of anti-tumor necrosis factor (anti-TNF) antibodies (*p* = 0.01) and nonsteroidal anti-inflammatory drugs (NSAIDs) (*p* = 0.026) in the axSpA group, and anti-interleukin 17 (anti-IL-17) antibodies were more commonly used in the axPsA group (*p* = 0.002), as shown in Table 1.

**Table 4:** Imaging findings according to phenotype

Variable	axSpA *n* = 33	axPsA *n* = 23	*p*
Presence of erosion, n %	18 (54,5%)	13 (56,5%)	0,884
Erosion score (SSS)	5,1 (± 8,24)	1,6 (± 3,8)	0,951
Presence of BF, n %	8 (24,2%)	2 (8,7%)	0,161
BF score (SSS)	1,5 (± 3,8)	0,5 (± 1,2)	0,119
Presence of FM, n %	15 (45,4%)	9 (39,1%)	0,638
FM score (SSS)	7,2 (± 11,1)	1,5 (± 3,2)	0,449
Presence of ankylosis, n %	11 (33,3%)	7 (30,4%)	0,819
Ankylosis score (SSS)	5,7 (± 7,8)	5,4 (± 7,7)	0,718
SSS total	19,6 (± 12,0)	9,0 (± 9,7)	0,068
Presence of BME, n%	15 (45,4%)	10 (43,5%)	0,884
SPARCC	3,8 (± 7,9)	3,54 (± 5,0)	0,913
mSASSS	16,9 (± 21,4)*	5,1 (± 8,2)**	0,981

*n = 43, **n = 15

*AxSpA* axial spondyloarthritis, *AxPsA* axial psoriatic arthritis, *BF* Backfill, *SSS* Sacroiliac Joint Structural Score, *FM* Fat Metaplasia, *BME* Bone Marrow Edema, *SPARCC* Spondyloarthritis Research Consortium of Canada Magnetic Resonance Imaging Index, *mSASSS* Modified Stoke Ankylosing Spondylitis Spine Score

### Difference between males and females

In our study, we observed notable differences related to sex. Men demonstrated higher scores in mSASSS, SSS, and FM score by SSS (Table 5). In the multivariate linear regression model with the dependent variable being SSS and the independent variables being sex, disease duration and HLA-B27 positivity, male sex (*p* = 0,002) and HLA-B27 (*p* = 0,014) were associated with SSS (Table 3). Particularly within the male subgroup, HLA-B27 carriers showed a higher SSS score compared to non-carriers (24 ± 12.4 vs. 13.5 ± 10.9; *p* = 0.013).

**Table 5:** Imaging examination data of the total sample according to sex

Variables	Male (*N* = 35)	Female (*N* = 21)	*p*
Presence of erosion, n %	19 (54,3%)	12 (57,1%)	0,835
Erosion score (SSS)	4,2 (± 5,6)	12,3 (± 18,8)	0,087
Presence of BF, n %	6 (17,1%)	4 (19,0%)	0,728
BF score (SSS)	1,1 (± 1,8)	8 (± 12,5)	0,859
Presence of FM, n %	21 (60,0%)	3 (14,2%)	**0**,**001**
FM score (SSS)	8,0 (12,5)	5,6 (± 8,3)	**0**,**001**
Presence of ankylosis, n %	14 (40,0%)	4 (19,0%)	0,143
Ankylosis score (SSS)	5,6 (± 8,31)	1,0 (± 1,73)	0,055
SSS	19,0 (± 8,5)	13,3 (± 17,8)	**0**,**001**
Presence of BME, n%	13 (37,1%)	12 (57,1%)	0,145
SPARCC	1,0 (± 2,4)	5,0 (± 8,6)	**0**,**039**
mSASSS	21,7 (± 19,3)*	10,4 (± 1,0)**	**0**,**008**

*n = 42, **n = 16

*AxSpA* axial spondyloarthritis, *AxPsA* axial psoriatic arthritis, *BF* Backfill, *SSS* Sacroiliac Joint Structural Score, *FM* Fat Metaplasia, *BME* Bone Marrow Edema, *SPARCC* Spondyloarthritis Research Consortium of Canada Magnetic Resonance Imaging Index, *mSASSS* Modified Stoke Ankylosing Spondylitis Spine Score. Values in bold indicate statistical significance with *p* < 0.05

On the other side, women exhibited a significantly elevated SPARCC score (Table 5). In the multivariate linear regression model, including SPARCC as dependent variable and sex, BMI and smoking as independent, only female sex (*p* = 0,035) was associated with SPARCC (Table 3). Women had a greater prevalence and fibromyalgia (*p* < 0.001), as detailed in Table 6.

**Table 6:** Clinical and demographic characteristics according to sex in the total sample

Variable	Male (*N* = 59)	Female (*N* = 35)	*p*
Age, years	51,3 (± 9,3)	38,6 (± 9,8)	0,413
axSpA (%, N)	59,7 (37)	40,3 (25)	0,5
axPsA (%,N)	68,8 (22)	31,2 (10)	0,5
BMI, kg/m²	30,4 (± 7,5)	28,3 (± 6,8)	0,324
Disease duration, years	16,8 (± 6,8)	7,3 (± 7,5)	0,073
Disease delay, years	10,8 (± 8,6)	5,67 (± 8,0)	0,534
ASDAS-CRP	2,7 (± 1,2)	3,7 (± 0,4)	0,592
BASDAI	4,3 (± 2,2)	4,9 (± 1,6)	0,093
BASFI	4,6 (± 2,7)	7,81 (± 13,9)	0,507
BASMI	4,6 (± 2,0)	7,53 (± 13,6)	0,395
Regular physical activity, n%	11 (18,6%)	8 (22,8%)	0,574
HLAB27 positive, n %	34 (57,6%)	17 (48,5%)	0,716
Manual labor, n %	18 30,5%)	5 (14,2%)	0,127
Smoking n%	6 (10,1%)	5 (14,2%)	0,629
Fibromyalgia, n %	5 (8,4%)	15 (42,8%)	**< 0**,**001**
Uveitis, n %	10 (16,9%)	9 (25,7%)	0,306
Inflammatory bowel disease, n %	1 (1,7%)	3 (8,5%)	0,137
Arthritis, n %	26 (44,1%)	17 (48,6%)	0,672
Enthesitis, n %	29 (49,1%)	15 (42,8%)	0,733
Dactylitis, n %	1 (1,7%)	3 (8,6%)	0,144

*axSpA* axial spondyloarthritis, *BMI* Body Mass Index, *BASDAI* Bath Ankylosing Spondylitis Disease Activity Index, *ASDAS-CRP* Axial Spondyloarthritis Disease Activity Score - C-Reactive Protein, *BASFI* Bath Ankylosing Spondylitis Functional Index, *BASMI* Bath Ankylosing Spondylitis Metrology Index. Values in bold indicates statistical significance with *p* < 0.05

## Discussion

Our results showed that, in the comparison of sacroiliac magnetic resonance imaging (MRI) between patients with spondyloarthritis with axial involvement, there were more differences related to sex than clinical phenotypes.

In our sample of 94 patients from southern Brazil, active disease was predominant (BASDAI > 4), specifically high disease activity (ASDAS-CRP > 2.1), with no difference between the groups. A Brazilian cohort study of 1492 patients with spondyloarthritis (SpA) reported a similar mean BASDAI (4.2), and like our study, found no difference between the diseases, reinforcing that the disease burden is very similar between the groups of axSpA [5]. There was a higher use of biologic agents (82.4%) compared to NSAIDs but with no difference between the groups. The use of NSAIDs was higher in the axSpA group (*p* = 0.026) and lower than the overall average in Latin American countries (68–98%), as shown in a recent meta-analysis [24]. The higher proportion of anti-IL-17 therapy use in axPsA (43.7%) and anti-TNF therapy use in axial SpA (66.1%) may reflect actual treatment recommendations for axial spondyloarthritis in Brazil, in which there is a preference for anti-IL-17 for severe psoriasis, more common in axPsA, and anti-TNF for other extra-musculoskeletal manifestations as uveitis and gastrointestinal disease, more common in axSpA [25].

There was no difference in radiographic damage in the spine assessed by mSASSS between the phenotypes, perhaps due to the limited number of available spinal radiographs for analysis, as studies suggest that ankylosing spondylitis has more severe radiographic involvement of the spine [26].

Regarding sacroiliac MRI, there was no difference in the findings of active or structural lesions between axSpA and axPsA, similar to another Brazilian study [27]. The mean SPARCC scores were relatively low and similar between the phenotypes, likely because the majority of patients were already under treatment when data was collected. Similar findings were observed in a study examining imaging in axial SpA, where most MRIs had low frequency of bone edema, although in that study [5], the authors did not evaluate structural lesions. Gensler et al. [28] compared MRIs between groups of non-radiographic axSpA with and without psoriasis and also did not observe differences in the prevalence of bone edema between the groups, only more asymmetrical sacroiliitis, lower HLA-B27 positivity, and older age in patients with psoriasis.

In radiographic evaluation, men had worse mSASSS, while age and manual labor were independent predictors of mSASSS. The association of worse radiographic damage with male sex is well-described in the literature [29–31]. It is also known that physically demanding jobs may amplify the potentiating effects of inflammation on bone formation in axial spondyloarthritis [32]. It is interesting to note that there was a correlation between mSASSS and BASMI, reinforcing the impact of radiographic progression on mobility, as previously reported by Protopopov et al., where higher mSASSS was associated with worse BASMI and BASFI scores [33].

In the MRI analysis, fat metaplasia was more prevalent in men, similar to another study that showed a 3.2 times higher association of this lesion in male patients [OR = 3.23, 95% CI (1.18, 9.53)] [34]. It is known that fat metaplasia is a predictor of ankylosis in spondyloarthritis [35], which justifies greater radiographic damage in male patients. Moreover, SSS was higher in the male group, in which sex and HLAB27 positivity were independent predictors. On the other side, SPARCC score was higher in the female group. A recent study with 379 patients with axial spondyloarthritis observed significant differences in sacroiliac MRI between men and women, showing better diagnostic performance for ankylosis, fat metaplasia, and erosions in men, while sclerosis and bone marrow edema were better markers for diagnosis in women [36].

Another interesting aspect is that in the present study, there was no correlation between disease activity scores and SPARCC, which has also been observed in another study [37]. This difference can be partially explained by the impact of structural damage and sensitization/fibromyalgia on disease activity scores, while MRI often reflects active inflammatory disease [38, 39].

SPARCC was positively correlated with the backfill score by SSS, which is expected since bone marrow edema is a predictor of structural lesions on MRI. Similarly, another study with 52 MRIs of patients with axial SpA observed that higher initial SPARCC scores were associated with greater longitudinal backfill progression [40].

HLA-B27 positivity was an independent predictor of structural damage (SSS score) and particularly in the male group, HLA-B27 positive patients had higher SSS than HLA-B27 negative ones. In a recent study in healthy individuals, being HLA-B27 positive impacted the extent of bone marrow edema (BME) on sacroiliac MRI only in men [41]. Extrapolating to spinal radiography data, the OASIS cohort [42] showed that HLA-B27 positive men, but not women, had significantly greater radiographic progression compared to HLA-B27 negative men. This reinforces HLA-B27 as not only a susceptibility factor but also a worse prognostic factor, particularly in men.

Patients with axSpA had a higher prevalence of HLA-B27 positivity compared to the axPsA group, as expected and shown in other studies [43]. Patients with axPsA were older and had a higher prevalence of peripheral arthritis. It is known that isolated axial involvement in psoriatic arthritis is rare (5%) [44] and the number of affected peripheral joints is a risk factor for axial involvement in psoriatic arthritis, unlike other spondyloarthritis conditions, where there is no apparent relationship between peripheral arthritis and axial involvement [45].

Although women showed numerically higher disease activity and functional metrics, there was no statistical significance compared to men, in contrast to a Portuguese cohort study [46] where female participants had a higher BASDAI score compared to our study (5.7 vs. 4.9) and statistically higher than in men. It is interesting to note that in our study, the mean BASMI score was numerically higher in women, although the literature describes that men have worse BASMI scores due to more severe radiographic damage [7].

The diagnosis of fibromyalgia was more common in the female group, a well-documented fact, as shown in a study that assessed diffuse pain in patients with spondyloarthritis, where women exhibited a two to three times higher probability of generalized axial pain (OR, 3.33; *p* = 0.007) and peripheral joint pain (OR, 2.34; *p* = 0.023) [47].

The present study is one of the few that compared sacroiliac MRI findings and the only one to compare the SSS between patients with axSpA and axPsA. The weaknesses include the sample size, transversal design, and the retrospective analysis of clinical and demographic data.

## Conclusion

Axial spondyloarthritis and axial psoriatic arthritis are diseases that differ in various clinical, epidemiological, and genetic aspects. However, they seem to have similar findings in the evaluation of sacroiliac MRI and spine radiographic damage. On the other hand, sex appears to be more relevant in the evaluation of axial spondyloarthritis through imaging, and this may have an impact on future diagnostic recommendations. Further studies with a larger number of patients are needed to confirm these findings.

## Data Availability

The data collected in this study, as well as the statistical analysis performed using the SPSS software, are available and can be promptly provided to interested parties upon request.

## Abbreviations

SpA: Spondyloarthritis
axSpA: Axial spondyloarthritis
axPsA: Axial psoriatic arthritis
AS: Ankylosing spondylitis
nr-axSpA: Non-radiographic axial spondyloarthritis
PsA: Psoriatic arthritis
nr-PsA: Non-radiographic psoriatic arthritis
MRI: Magnetic resonance imaging
BME: Bone marrow edema
ER: Erosion
FAT: Fat metaplasia
SPARCC: Spondyloarthritis Research Consortium of Canada Magnetic Resonance Imaging Index
SSS: Sacroiliac Joint Structural Score
MSASSS: Modified Stoke Ankylosing Spondylitis Spine Score
ASAS: Assessment of SpondyloArthritis international Society
CASPAR: Classification Criteria for Psoriatic Arthritis
OMERACT: Outcome Measures for Arthritis in Clinical Trials
BMI: Body Mass Index
BASDAI: Bath Ankylosing Spondylitis Disease Activity Index
ASDAS: Axial Spondyloarthritis Disease Activity Score
ASDAS-CRP: Axial Spondyloarthritis Disease Activity Score C Reactive protein
BASFI: Bath Ankylosing Spondylitis Functional Index
BASMI: Bath Ankylosing Spondylitis Metrology Index
HLA-B27: Human Leukocyte Antigen B27
STIR: Short Time Inversion Recovery
T2: Transverse relaxation time
T1-WSE-MRI: T1 weighted spin echo magnetic resonance imaging
ICC: Intraclass correlation coefficient
anti-TNF: Anti-tumor necrosis factor
NSAID: Non-steroidal anti-inflammatory drug
anti-IL-17: Anti-interleukin 17
DAPSA: Disease Activity Index for Psoriatic Arthritis
BSA: Body Surface Area
PASI: Psoriasis Area Severity Index
